# Ideal Binocular Disparity Detectors Learned Using Independent Subspace Analysis on Binocular Natural Image Pairs

**DOI:** 10.1371/journal.pone.0150117

**Published:** 2016-03-16

**Authors:** David W. Hunter, Paul B. Hibbard

**Affiliations:** 1 School of Psychology and Neuroscience, University of St Andrews, St Andrews, United Kingdom; 2 Department of Psychology, University of Essex, Colchester, United Kingdom; University of Montreal, CANADA

## Abstract

An influential theory of mammalian vision, known as the efficient coding hypothesis, holds that early stages in the visual cortex attempts to form an efficient coding of ecologically valid stimuli. Although numerous authors have successfully modelled some aspects of early vision mathematically, closer inspection has found substantial discrepancies between the predictions of some of these models and observations of neurons in the visual cortex. In particular analysis of linear-non-linear models of simple-cells using Independent Component Analysis has found a strong bias towards features on the horoptor. In order to investigate the link between the information content of binocular images, mathematical models of complex cells and physiological recordings, we applied Independent Subspace Analysis to binocular image patches in order to learn a set of complex-cell-like models. We found that these complex-cell-like models exhibited a wide range of binocular disparity-discriminability, although only a minority exhibited high binocular discrimination scores. However, in common with the linear-non-linear model case we found that feature detection was limited to the horoptor suggesting that current mathematical models are limited in their ability to explain the functionality of the visual cortex.

## Introduction

### Binocular disparity detection

Many animals view the world through two eyes. Although two views of the world confer advantages such as a wider field of view, greater visual acuity in areas of overlapping fields of view and a perception of depth from binocular disparities, calculation of a single cyclopean view from two inputs is non-trivial. In order to create a single fused percept, and to estimate binocular disparity, corresponding features in both views must be matched. These features can vary in orientation, shape, and size, as well as their locations in the two eyes. The standard model of this process proposes that disparity is determined by cross-correlation of the visual signals from the left and right views [1, 2]. This idea of calculating a local cross-correlation has been linked to the activity of simple and complex cells in the primary visual cortex through the binocular energy model [3, 4].

#### Properties of an ideal disparity detector

Ohzawa et al. [4] argued that an ideal disparity detector should have the following three properties. Firstly, its disparity tuning should be narrower than the size of the neuron’s receptive field. Secondly, the preferred disparity should be constant for all stimulus positions within the receptive field. Thirdly, incorrect contrast polarity matches (in which a feature is brighter than the background in one eye, but darker than the background in the other) should be ineffective at producing a response for stimuli at the detector’s preferred disparity. This latter property would allow the neuron to implement the similarity constraint (that only similar features, in this case those having the same contrast polarity, should be matched [5,6]).

**Fig 1:**
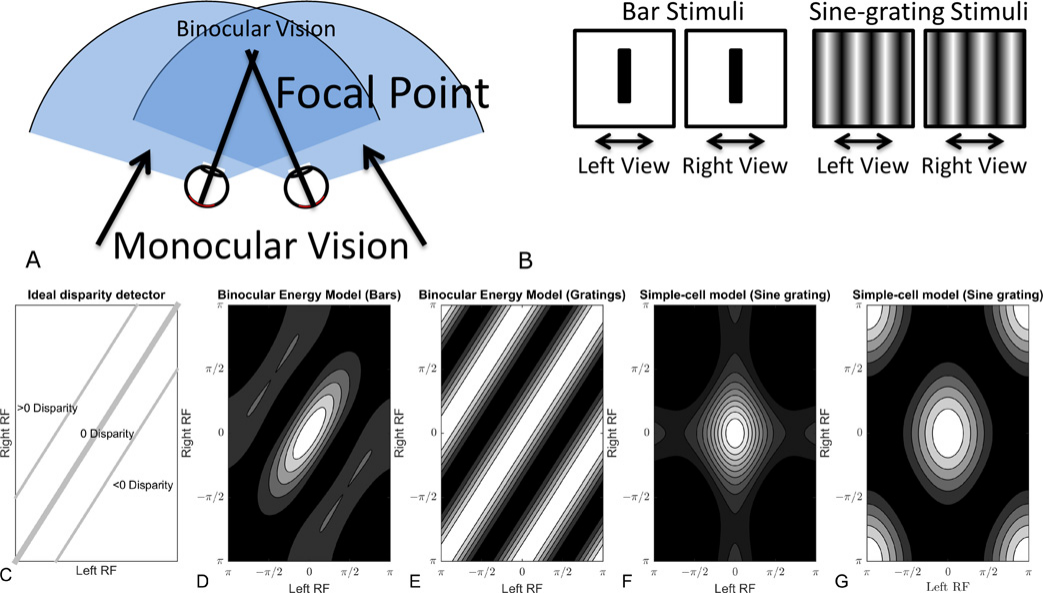
Properties of different models of binocular integration. The top row shows examples of bar and sine-grating stimuli (B). Subplot C, an ideal disparity detector responds strongly to a particular disparity and weakly elsewhere. Lines of equal disparity lie on the diagonal with zero disparity (shown as the thick-line) on the main diagonal. D&E, responses of the standard binocular energy model (21) to bar (D) and sine-grating (E) type stimuli. As before, zero disparities lie on the main diagonal. The strongest responses lie on the diagonal where disparities are zero, however strong responses also appear on sidebands at disparities of ±π. F&G, responses of simple-cell models to bar (F) and sine-grating (G) stimuli. Energy is concentrated in locations corresponding to specific combinations of positions in the receptive fields. Disparity in simple-cells is confounded with local spatial position.

These properties of an ideal disparity detector are summarised in Fig 1, showing how the response of a neuron to a simple bar stimulus or a sine grating might be affected by the position (or phase) of the stimulus in each eye. Fig 1 (far left) shows the ideal response of a neuron tuned to zero disparity, with the position of the stimulus in the left- and right-eyes’ images on the horizontal and vertical axes, respectively. This idealised neuron responds strongly when the stimulus is presented at the same location in each eye, regardless of the actual location. This creates a strong response along the diagonal. Fig 1C also shows an idealised neuron tuned to a non-zero disparity. Again, strong responses occur along a diagonal line, but in this case it is shifted rightwards, indicating that the neuron responds most strongly for a specific offset of the stimulus between the two eye’s images.

### Binocular cells in the visual cortex

The responses of many cells in V1 are affected by stimuli presented to both eyes. Some cells have a clearly defined receptive field for each eye, such that an appropriate stimulus presented to either the left or the right eye will produce a response. To a first approximation, the overall response of the cell is then the sum of the responses to the left- and right-eyes’ stimuli. Other cells can be considered monocular in that an appropriate stimulus must be presented to a particular eye in order to evoke a response, and no response is evoked if a stimulus is only presented to the other eye. Even in this case, however, there can be clear binocular interactions, in that the response to a stimulus in one eye is modulated by the stimulus presented to the other eye [7].

For cells with a receptive field in each eye, disparity tuning occurs through both position shifts and phase shifts of the receptive fields. A position shift refers to the situation in which the receptive fields are in different locations in the two eyes. A phase shift refers to the situation in which the shapes of the receptive fields (the spatial arrangement of excitatory and inhibitory lobes) are different. Typically, disparity-tuned neurons in V1 show a combination of position and phase shifts [8, 9].

#### Disparity tuning of simple and complex cells in V1

Computational models of binocular complex-cells have been derived from the *standard energy model* of Adelson and Bergen [10] by Ohzawa et al.[4], Qian & Zhu[11] and Fleet[3]. The *binocular energy model* consists of a two layer hierarchical network; the responses of a pair of binocular linear filters in quadrature phase are squared and additively combined to produce the energy response.

The standard binocular energy model estimates disparity from the relative energies of features in the two binocular views. This model[3] can be conceptualised as a complex-cell integrating over the responses of simple linear-non-linear monocular subunits. The binocular energy model is defined as:

$$bem={\left(R_{L}+R_{R}\right)}^{2}+{\left(I_{L}+I_{R}\right)}^{2}$$ (1)

Where the result of weighting the left input image by an even Gabor function is denoted as $R_{L}$ and with an odd Gabor function as $I_{L}$. We denote the responses from the right input image similarly. In this equation each of $R_{L}R_{R},I_{L},I_{R}$, is considered a subunit in analogy to the subunits observed in complex-cells in the visual cortex. Fleet et al [3] showed that the binocular energy response can be expressed as:

$$bem=R_{L}^{2}+I_{L}^{2}+R_{R}^{2}+I_{R}^{2}+2\sqrt{{\left(R_{L}+I_{L}\right)}^{2}{\left(R_{R}+I_{R}\right)}^{2}}\cos\left(\Delta \phi \right)$$ (2)

where Δ*ϕ* is the binocular phase difference. This can be rewritten as

$$\cos\left(\Delta \phi \right)=\frac{{\left(R_{L}+R_{R}\right)}^{2}+{\left(I_{L}+I_{R}\right)}^{2}-R_{L}^{2}-R_{R}^{2}-I_{L}^{2}-I_{R}^{2}}{2\sqrt{{\left(R_{L}+I_{L}\right)}^{2}+{\left(R_{R}+I_{R}\right)}^{2}}}$$ (3)

The local binocular phase difference Δ*ϕ* can thus be calculated by combining the binocular energy response with monocular energy responses. Fleet et al. [3] showed that the binocular energy responses are related to a point-wise cross-correlation between of the filtered left and right eyes’ images.

### Efficient coding of visual information

Barlow suggested that an efficient coding of ecologically valid visual stimuli could explain the receptive field patterns of neurons in the visual cortex[12]. Such a coding would need to balance a trade-off between the energy of responses evoked by a stimulus and the number of neurons used in the coding. The highly peaked nature of frequency and orientation distributions in natural images has led to the hypothesis that an efficient coding of visual stimuli would be sparse. By eliciting a few large amplitude responses to each stimulus such a coding would be maximally energy efficient.

#### Sparse coding and ICA as a model of receptive field properties of V1

By imposing a highly kurtotic prior on neural responses [13], or simply maximising the response kurtosis [14], filters can be learned from natural images that resemble the Gabor-like patterns of receptive fields of simple V1 cells. Similar results have been found for binocular natural images; maximising kurtosis [15] and information [16] have both been found to produce filters similar to binocular receptive fields of simple-cells in V1. These methods all belong to the class of algorithms known as Independent Component Analysis [17, 18]. However Ringach [19] observed important differences between the structure of receptive fields of simple-cells in macaque and the structure of linear components learned using ICA. In particular ICA components tended to be significantly more narrowband frequency and orientation tuned than cells observed in the visual cortex[19].

#### Independent Component Analysis for binocular images

Independent Component Analysis of binocular image patches has found binocular pairs of Gabor-like components [16, 20, 21]. These components exhibit a mix of both monocular (most component energy in one view) and binocular responses. In general, the receptive fields of the binocular components had a very similar orientation and spatial frequency in the left and right views. The components exhibited a range of binocular phase [16, 20] and position disparities [21]. The distributions of position disparities were dependent on the wavelength of the component’s carrier function and phase disparities were biased towards correlated (TE) and anti-correlated (TI) components. These phase and position disparities combined (in each component) results in total disparities that lie on integer and half integer multiples of the wavelength of the component [21].

#### Correspondence between ICA results and the properties of cortical neurons

The accuracy with which Independent Component Analysis replicates the receptive fields of simple-cells in V1 has been questioned. The components learned by ICA tend to have narrower spatial frequency and orientation bandwidths than the receptive fields of V1 simple cells [19]. Also, the proportion of anti-correlated components found using binocular ICA was higher than that observed in macaque V1 [21]. Notably, although ICA components coded for a wide range of both phase and position disparities when measured individually, when phase and position where combined into a single overall disparity measure, disparities coded for by ICA components were restricted to integer and half integer multiples of the component’s wavelength[21]. This bias towards particular disparities has not been found in the visual cortex of mammals [8, 9, 22]. To address some of these discrepancies, there has been some work to improve statistical models of vision beyond ICA. For example, Olshausen proposed that imposing a sparsity inducing constraint based on the *L*_1_ norm (the sum of the absolute responses across the population) on the learning process produced components with the broadband tunings observed in V1 [23, 24].

#### Beyond linear models

A number of authors have also extended the simple linear models of natural image statistics to nonlinear, complex-cell models. The underlying principle of these methods is to distinguish between two types of interactions between component responses. In simple linear-nonlinear models, interactions are considered to be mostly inhibitory, resulting in a sparse set of responses. However, the responses of the subunits of complex cells in the cortex are not thought to interact in that way. Rather, most authors have considered the responses of complex cell models to be decorrelated, and have modelled them using a Gaussian distribution (also known as an *L*_2_ norm)[11, 15, 25–29]. Between complex-cells, the relationship between responses is assumed to be sparse[15, 25]. Hyvärinen and Hoyer (2000) formulated an extension to ICA, Independent Subspace Analysis, that learns a set of complex-cell-like components from natural image patches by fixing an orthogonal *L*_2_ norm (the square root of the sum of squared responses) between the linear subunits of complex cells, while maximising response kurtosis between complex cell components. Provided the distribution underlying the data is sparse, maximising response kurtosis will produce a model that produces sparse responses[18].

This extension of ICA is important in providing the invariance to phase and position that is a requirement of an ideal disparity detector [4]. The responses of the linear filters generated by ICA depend on the relationship between the phase of the filter, and of the input image. This phase dependence is similar to that of simple cells in the visual cortex, and to the first-stage filters in the binocular energy model. In the energy model, phase invariance is achieved by summing the squared responses of linear filters that are tuned to different phases[4]. Independent Subspace Analysis, in which the subspace response is the sum of the squared responses of the individual subunits, has the potential to learn phase invariance in a similar way. Indeed, using their ISA model, Hyvärinen and Hoyer learned complex-cell-like models that exhibited phase or position invariance, through a set of linear components with similar frequency and orientation, but shifted phase and position[15]. Thus, while the linear components learned through ICA are not suitable candidates as ideal disparity detectors, the complex-cell-like models learned by ISA have the potential to perform this role.

Burge and Geisler [30] developed a supervised learning model specifically designed to learn to estimate disparity from binocular images. Rather than attempt to learn an encoding that spanned the full range of observed stimuli, Burge and Geisler learned encodings optimised specifically for the task of estimating disparity. Burge and Geisler’s model summed over both linear and squared subunits and allowed for second-order interactions between subunits. By taking into account complex linear and nonlinear interactions, Burge and Geisler’s model allowed for a much narrower disparity tuning than the standard energy model[30].

The binocular energy model has been shown to be a highly effective model of complex binocular cells in the mammalian visual cortex[4,7,10,31–36]. Similarly, statistical models based on the energy model have been shown to be capable of unsupervised learning of spatially invariant complex-cell models of monocular images [15, 25] and supervised learning of neural networks to estimate binocular disparity[30]. In this study we compared complex-cell models learned from binocular natural images to the properties of an ideal disparity detector. We used ISA in order to learn models that exhibit the phase-invariance that characterises the responses of complex cells, and is a necessary component of ideal disparity detectors.

## Methods and Materials

We performed Independent Subspace Analysis [15] on rectangular image patches cut from binocular image pairs [37]. This analysis produced a set of non-linear models whose responses to binocular image stimuli was explored using responses to sine-grating and bar stimuli. Finally we used the Disparity Discrimination Index [9] to determine how much of the variance in complex-cell model responses could be explained by the disparity of the stimuli.

### Data-set

We performed our analysis on a set of binocular photographs of various scenes, designed to approximate binocular vision. This data-set was previously described in [37], for clarity we repeat the setup here. A set of binocular images was captured using a purpose built platform housing two Nikon Coolpix 4500 digital cameras. The platform allowed the inter-camera separation along the horizontal axis, and the cameras’ orientations around the vertical axis, to be manipulated. This setup approximated the interocular distance between human eyes. Independent rotation about the vertical axis afforded one degree of freedom of movement of each view, allowing simulation of vergence on a focal point within the scene. This setup assumes that differences in elevation and cyclovergence in each eye is zero. In all scenes the cameras were separated by 65mm. The cameras were oriented such that the same point in each scene was centred in each camera image. This approximately mimics the expected fixation strategy a human would adopt to focus on an object directly in front of them.

The scenes varied in both composition and range of disparities contained. A number of indoor scenes consisting of a range of mixed fruit and vegetables were captured on a light-table. A range of outdoor scenes were captured in the area in and around the town of St Andrews in Scotland, these included a range of beach and woodland scenes. The full set of images collected can be downloaded from www.github.com/DavidWilliamHunter/Bivis.

The captured images were calibrated to account for the lens and colour characteristics of the camera. We used the Camera Calibration Toolbox for Matlab [38] to calibrate for the optics of the camera’s lenses, and transformed the images such that they approximated an image taken with a perfect ‘pinhole-camera’. A consequence of this transformation is that we can describe pixels in terms of the visual angle they subtend. The images were captured at a resolution of 1600 x 1200 pixels prior to calibration and reduced and calibrated to 1201 x 1201 pixels, where each pixel covered 1 arc minute of visual angle. The images were converted to CIE LAB values [39] using colour patches captured from a Macbeth Colorchecker DC chart to establish the colour characteristics of the camera.

### Learning the complex cell model

#### Pre-processing

We sampled 500,000 25 by 25 pixel patches from the binocular image set. The images were prepossessed using the methods of [21], reprised here for clarity. Pre-processing involves two steps that are necessary for calculation of ISA components [15] and have similarities with early stages of visual processing in the retina and LGN. Retinal ganglion cells are known to have centre-surround response profiles[40] that de-correlate visual input [41]. The retina also plays a role in gain control[42]. For more details on the implications of the pre-processing see [21].

***x***_*i*_ is the i^th^ vectorised image patch. $x_{i}^{\left(l\right)}$ is the i^th^ image patch from left view and $x_{i}^{\left(r\right)}$ is the i^th^ image from the right view. As we are primarily interested in local structure rather than overall luminance we centre and normalise image patches separately for each eye. We centred the luminance of each image patch separately by subtracting the patch mean luminance.

$${\bar{x}}_{i}^{\left(l\right)}=x_{i}^{\left(l\right)}-\langle x_{i}^{\left(l\right)}\rangle$$ (4)

where ⟨*x*⟩ denotes the mean of *x*. The same method was applied to the right view patches. We normalise the patches using,

$${\dot{x}}_{i}=\left[\frac{{\bar{x}}_{i}^{\left(l\right)}}{\left\|{\bar{x}}_{i}^{\left(l\right)}\right\|},\frac{{\bar{x}}_{i}^{\left(r\right)}}{\left\|{\bar{x}}_{i}^{\left(r\right)}\right\|}\right]$$ (5)

As it is a requirement of ISA that the samples are of unit length we normalise the whole vector again.

$$x_{i}=\frac{{\dot{x}}_{i}}{\left\|{\dot{x}}_{i}\right\|}$$ (6)

The vectors ***x***_*i*_ are concatenated into a matrix *X* such that *X* is an *N* by *M* matrix where *N* is the number of image patches and *M* is the number of pixels per patch.

A requirement of the ISA algorithm we are using is that the data are whitened using Principal Component Analysis prior to processing[15].

### Independent Subspace Analysis

The Independent Subspace Analysis algorithm was developed by Hyvärinen and Hoyer[15], in this section we briefly summarise the main features of their algorithm. The patch set *X* can be expressed as a linear combination of feature components.

X=AW (7)

where the matrix *A* is the feature components and *W* the set of weights. If *A* is restricted to the set of orthonormal basis functions then Eq 7 is invertible *A* = *XW*^−1^ as *W* is orthonormal, the inverse is also the transpose *W*^−1^ = *W*^*T*^.

In standard Independent Component Analysis a set of basis vectors (*W*) is learned such that (*A*) has a sparse leptokurtic distribution. This is achieved by maximising the non-Gaussianity of *A* using a non-linear function.

g(AW) (8)

where *g* is a non-linear function, in standard FastICA *g* is e.g. *tanh x* [18]. Subspace Component Analysis divides the components into sets of two, the norms of these sets is expected to be leptokurtic. In ISA the norm of a subspace is defined as:

$$\sqrt{\sum_{j=1}^{N}{\left\langle w_{j},x_{i}\right\rangle }^{2}}$$ (9)

**Fig 2:**
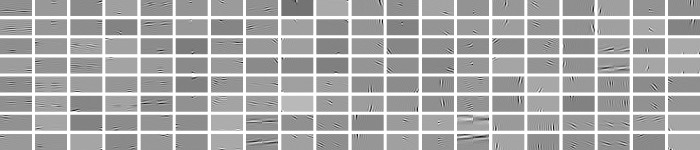
Example components learned using ISA. Each component is shown as a rectangular pair, the left half of each pair shows the receptive field (25 by 25 pixels square) in the left view, the right half of the rectangle shows the right view’s receptive field. Light areas of receptive field are excitatory and black areas inhibitory, grey areas are neural.

That is, the square root of the sum of squared responses. This norm has similarities with the *L*_2_ norm, due to the sum of squares term. Responses within the subspace have a Gaussian profile, and the square root term gives the responses between the subspaces a sparse profile. ISA components are solved using the method of maximum likelihood[16]. Examples of components learned using ISA are shown in Fig 2.

### Complex-cell Models

**Fig 3:**
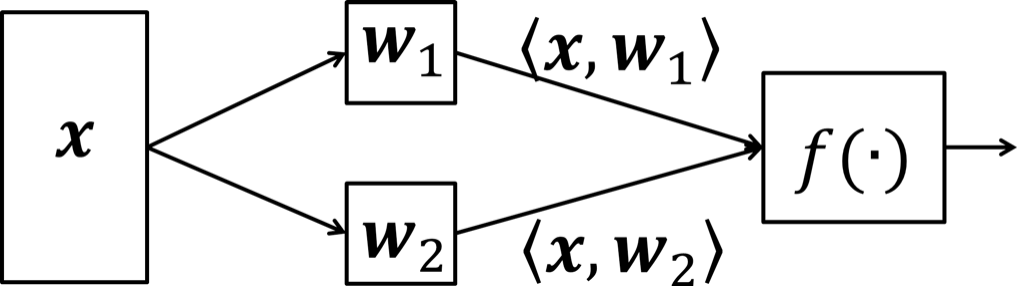
A two layer model of a complex neuron. The image (***x***) is filtered using a set of ***n*** basis functions (***w***_**1…*n***_) and combined into a single response by the non-linear function, ***f***.

Complex neurons are modelled in the manner of the standard binocular energy model [3] by combining the responses of two linear subunits by summation and applying a non-linear squaring function. A diagram of the complex neuron model can be found in Fig 3.

We trained an ISA model on 500,000 binocular image patches sampled from all 139 binocular image pairs in the dataset. The model consisted of 200 subspaces, each containing two subunits, resulting in 400 binocular components in total. Complex neurons were derived from the components of the subspace using the methods described above (the two linear components in each subspace are ***w***_1_ and ***w***_2_). Complex-cell models were modelled using the two linear subunits as

$$\sum_{i=1}^{2}{\left\langle x,w_{i}\right\rangle }^{2}$$ (10)

## Results

### Phase-phase plots: responses to sine-gratings

We used a phase-phase plot to examine the interaction between model responses and the phase of the stimuli in each view. This plot shows the interaction of the neuron’s responses to sine-gratings of different phases in each eye as a two-dimensional heatmap, each cell corresponding to a particular combination of phases. The frequency and orientation of the phase-phase plot are chosen such that the maximum response in the phase-phase range is maximised. A phase-phase plot is useful for determining responses to phase disparity in a binocular image. Example phase-phase plots of an ideal locally position invariant zero disparity detector can be seen in Fig 1C. Features in the binocular view are at zero disparity when they appear in the same location within the receptive fields in both views, thus zero disparities are found on the main diagonal in each plot. Disparities other than zero are found on a line offset from the main diagonal (as shown in Fig 1). The responses of the binocular energy model[21] to sine-gratings are found in Fig 1E. As with the ideal detector the strongest responses are found on the main diagonal indicating that the binocular energy model is strongly tuned to detect disparities of zero. There are also side-bands shifted by *π* as energy responses to sine gratings shifted in phase by *π* radians are indistinguishable from the original. Fig 1G far right, shows the response of a simple linear-non-linear model to the same sine-grating stimuli. The linear-non-linear model responds strongly only to particular combinations of phase in each view, thus disparity is confounded with local position.

**Fig 4:**
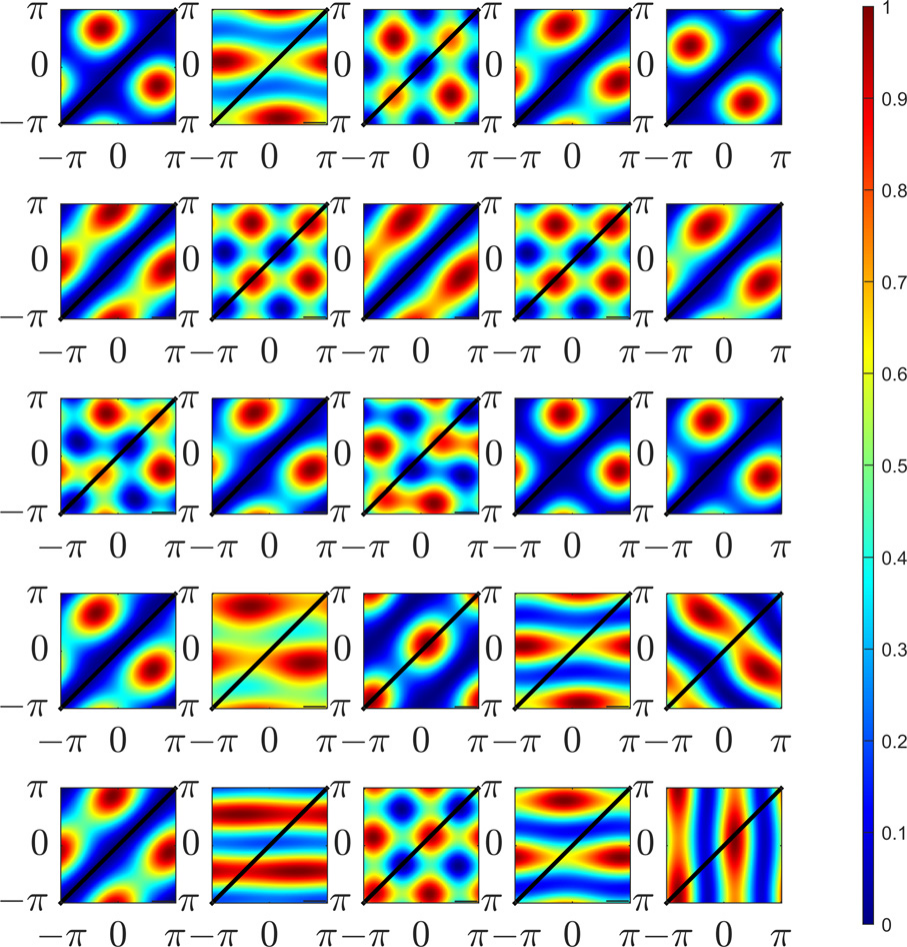
Example responses to binocular sine-gratings for 25 individual complex models learned using ISA. The models consist of two linear subunits combined in a sum of squares manner. The phase of the left view sine-grating is shown on the horizontal axis, the right view phase is shown on the horizontal axis. The phases in both views vary between −***π*** and ***π***. 100 samples were taken within this range for each view resulting in a 100x100 response map. Locations of equal disparity lie on diagonal lines, locations of zero-disparity are shown as a black line.

The frequencies and orientations of the simple-cell units were determined by fitting Gabor functions to the individual ISA sub-components as described in detail elsewhere[15]. Sine-gratings at the mean frequency and (angular) mean orientation of the sub-components were generated at varying phases. Binocular pairs of sine-gratings were presented to the complex-cell models; the phases were varied separately in each of the left/right views in order to generate phase disparity. The phases of the left right views were varied independently between −*π* and *π* to produce a 2D response map of the complex cells. Examples of the response maps to complex cells learned using ISA are shown in Fig 4. Locations in the response map of equal disparity lie along the diagonal. Zero disparity sine-gratings lie on the main diagonal from [−*π*,−*π*] to [*π*,*π*].

**Fig 5:**
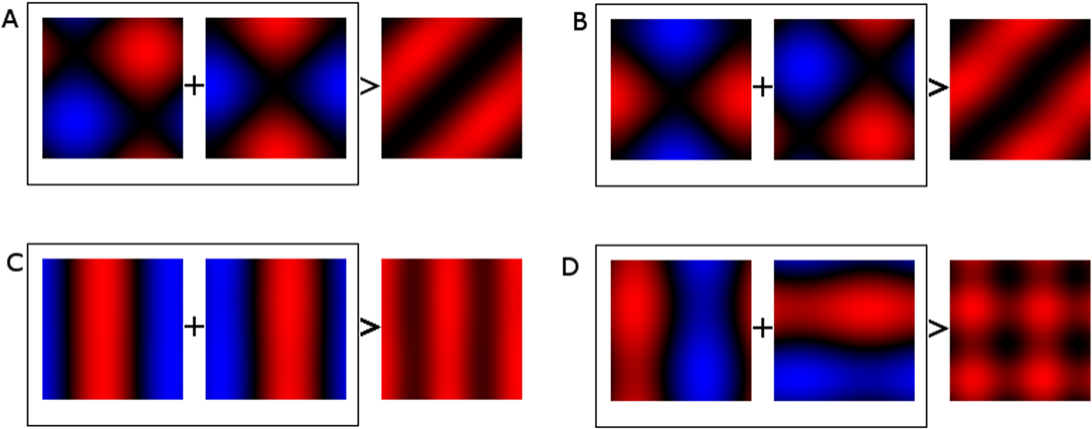
Extended examples of complex ISA models’ responses to sine-gratings. Four complex model responses are shown as combinations of two linear subunits. The two left-hand sine-grating plots show the simple linear responses of individual subunits to sine-gratings varying between–π and π in each view. The right gratings (after the >) show the complex model response (sum of squares of the subunits). The complex models are chosen to illustrate different ‘types’ of complex cell model, A & B show models with high binocular disparity discrimination scores (see DDI section). In both models the sub-units exhibit strong binocular tuning in phase, resulting in a strong response to particular left/right phase combinations and low responses elsewhere. Phase separation of approximately π⁄2 between the two sub-units results in a quadrature pairing and consistently strong response to a particular disparity. C & D show models with low DDI. In model C, both sub-units are monocular (responses modulated only one eye) resulting in a monocular complex cell. Model C is not phase invariant however the DDI index is not sensitive to monocular phase invariance, other monocular phase invariant models may exist. Model D also shows monocular sub-units, however in contrast to model C the sub-unit are differently monocular in each eye. Model D does not show any invariance in phase and appears to be specialised to detect particular phase combinations.

Individual sub-components are tuned to detect a particular phase in each view (not necessarily the same in each view) and thus produce blob-like structures. These components are tuned to detect a particular phase disparity at a particular phase. Complex cell models combine responses of individual sub-units. An ideal complex-cell model will have a uniform response to a particular disparity irrespective of phase, This results in a response map with the peak lying on a diagonal line on the sine-grating response plot. To illustrate these responses in detail, further examples of ISA complex model responses, together with their subunit responses, are shown in Fig 5. Examples of three types of complex-cell models learned using ISA are shown. Fig 5A and 5B show examples of complex-cell models strongly tuned to detect disparity, the maximum excitatory responses (red bands) are found along diagonal lines of equal disparity, although in both cases the bands are shifted from the main diagonal indicating a preference for non-zero disparity in both models. Fig 5C shows a monocular cell, the strongest responses lie in vertical bands indicating that the phase in right view (vertical axis) has little effect on the response of the model. Fig 5D shows an example of a model tuned to detect particular phases in each view, in this case the subunits are both monocular, the left subunit mostly responds to stimuli in the left eye (relatively small modulation of responses due to phase in the right view), the right subunit mostly responds to stimuli in the right eye.

### Responses to bar stimuli

**Fig 6:**
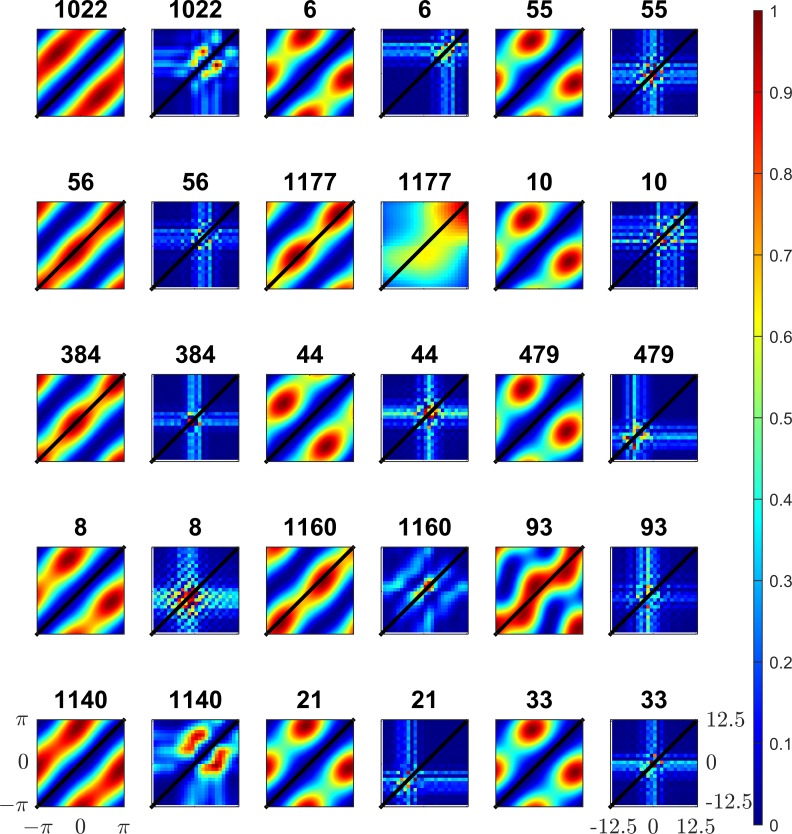
Responses of complex cell models to and both bar and sine-grating stimuli. The responses of 15 models (chosen for having high DDI) to both sine-grating (columns 1,3,5) and bar stimuli (columns 2,4,6) are shown. The model number is shown above the plot. Positions of zero disparity in both the sine-grating and bar plot are shown as a black line. As with previous Figs in models with high DDI strong responses lie predominantly on lines of constant disparity on the response to sine-gratings. Responses to bar stimuli are more localised as bar stimuli are sensitive to RF size. As with responses to sine-grating strong responses of high DDI complex cells to bar stimuli lie predominantly on lines of equal disparity.

Sine-gratings are useful stimuli for isolating responses to a particular frequency over a range of phases however they cover the entire field of view and so do not isolate the size of the receptive field [4, 7, 31, 35, 43–46]. Bar stimuli presented to each eye can be localised within the receptive field and thus can explore the bounds of the receptive fields. As with sine-gratings, we exposed our model complex cells to binocular bar stimuli with bar width set to half the wavelength of each complex cell model’s sub-units and infinite bar height, the orientation of the bar matched the mean sub-unit orientation in each model. Bar stimuli were presented to each of the left/right views at a range of shifts orthogonal to the bar orientation. These shifts simulate binocular disparity, if the shift is identical in each view then the stimuli are at zero disparity, otherwise the disparity is the difference between the shifts in each view. The stimuli presented to each model covered a range of -12.5 to 12.5 pixel shifts in both views. Response heatmaps are shown in Fig 6, as before the line marking zero disparity shifts lies on the diagonal (bottom-left to top-right).

As before, the pattern of responses reveals information about what the model is tuned to detect. The size of the receptive field is clearly visible as the models only respond to a bar stimulus when it lies within the receptive field. As before the complex-cell models can be categorised into distinct subsets; monocular cells are modulated only by the location of the bar in one view, binocular cells are modulated by the bar location in both views. In Fig 6 the monocular cell type can be identified by response maps containing only horizontal and vertical stripes. Notably, these stripes are collocated or located adjacent to each other in the response-map; this shows that the receptive fields of the sub-units are located in spatially similar areas. Binocular complex cell models are modulated by the position of the bar in both left and right views resulting in a cross-like structure in the response-map. The peaks of the responses lie along diagonal lines of equal disparity as would be expected of binocularly selective-cells.

Fig 6 shows the responses to both bar and sine-grating stimuli, for complex cell models that were chosen as being strongly selective to the disparity of sine-grating stimuli. As with responses to sine-gratings, these models generate strong responses to particular disparities. Unlike for sine-gratings, these responses are localised to a small area of the plot; this is due to the size of the receptive fields. The cells are tuned to detect a wide range of disparities, as shown by the number of models with strong responses off the central zero-disparity diagonal. The responses generated by bar stimuli resemble the responses of binocular tuned neurons in the striate cortex in cats [35]. Models 1022 and 1140 in Fig 6 strikingly resemble cell recordings (see [35] Fig 3). This model exhibits two features: strong responses for particular position disparities and slightly weaker (but still clear) responses to monocular stimuli, as shown by the vertical and horizontal bars in almost all response plots.

### Disparity Discrimination Index

**Fig 7:**
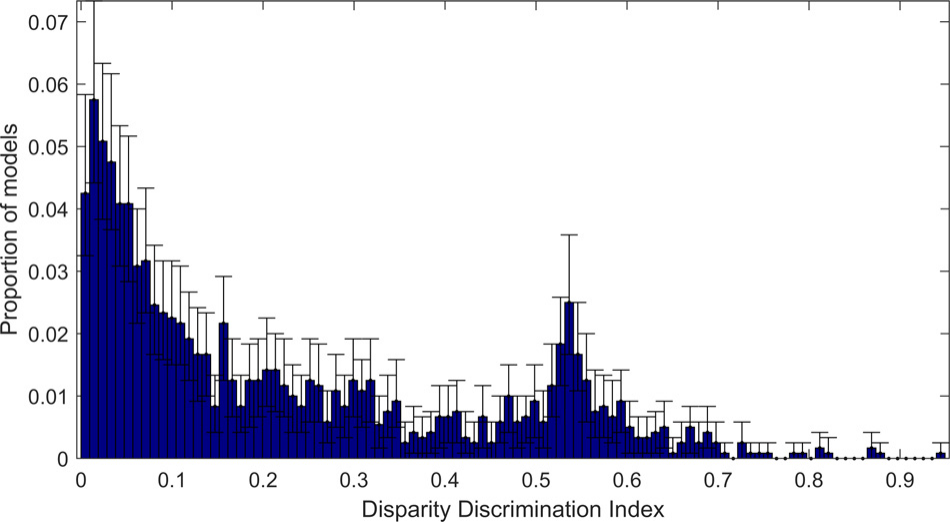
Bootstrapped distribution of Disparity Discrimination Index for complex models learned using ISA. The blue bars show the median of the bootstrapped results for each bin, the error bars show the 95% Confidence Intervals for calculated using 100 bootstraps. The complex cells exhibit a wide range of distributions from non-disparity selective models with low DDI to very high disparity selectivity with high DDI.

The heatmaps of the responses to both bar and sine-grating stimuli allow us to visualise the pattern of the model neuron’s responses to visual stimuli. However visualisation alone does not allow us to quantify the selectivity of the model neuron to disparity. In order to measure disparity selectivity, we used the Disparity Discrimination Index of Prince et al. [8, 9]. The Disparity Discrimination Index (‘DDI’) estimates the proportion of variation in the response that is explained by disparity. We computed the DDI for each complex model learned using ISA, using the responses of the model to sine-grating stimuli. As before the frequency and orientation of the sine-gratings were selected separately for each model such that model responses were maximised. A histogram of model DDIs are shown in Fig 7. The vast majority of complex-cell models have low DDI and therefore are only weakly (at best) selective for disparity. 20% of the complex-cell models have middling to high disparity selectivity, a DDI of higher than 0.47. The 95 percentile of the DDIs is 0.60. Although only a minority of the models are strongly binocularly selective they show an extremely strong selectivity, the maximum is a DDI 0.95, almost all the variance in responses is explained by disparity selectivity.

### Response function Symmetry

The symmetry of variations in responses of neurons to stimuli of varying disparity has been used by a number of authors to classify these cells in terms of their binocular responses[47]. Tuned Excitatory (TE) neurons, which respond most strongly to stimuli on the horoptor (zero disparity), will have an even symmetric disparity response function, neurons that are most inhibited by stimuli on the horoptor are also even symmetric with a response function roughly inverted in comparison to a TE cell, these are labelled Tuned Inhibitory. Odd symmetric disparity response functions indicate a neuron tuned to detect stimuli that lie either in front of (Near), or behind (Far), the horoptor. Most authors have found a strong bias towards even symmetric (TE/TI) neurons compared to odd symmetric neurons (Near/Far)[8, 47, 48]. A representative result from Prince et al. found 38% TE, 16%TI, 21% Near and 25% Far.[8].

**Fig 8:**
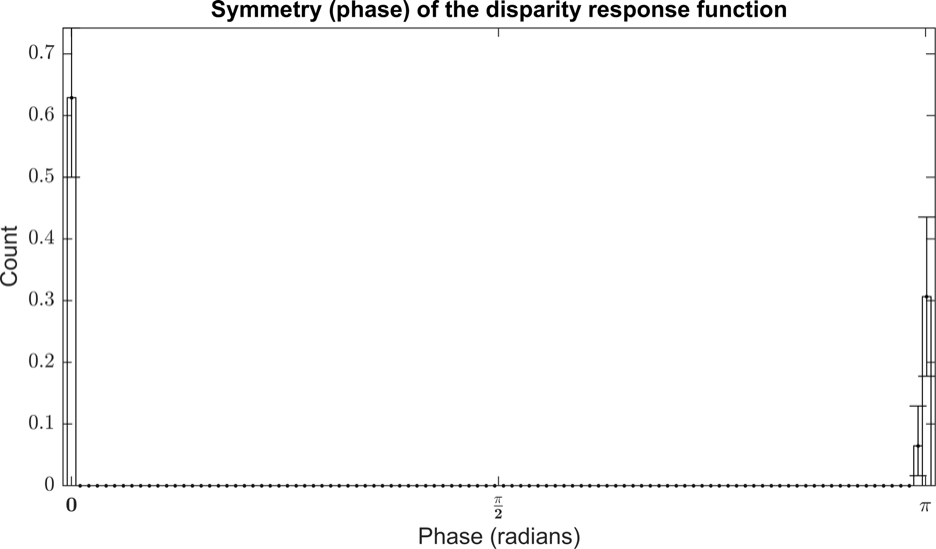
Bootstrapped histogram of the phase of the disparity response functions for each complex model with a DDI greater than 0.6. Phases of 0 and π radians show even-symmetric disparity functions and indicate TE (0) and TI (π). All complex cell model with a DDI greater than 0.6 show TI and TE cell exclusively. Cells at lower DDI are not disparity tuned.

We measured the response symmetry using the phase of a sine wave function fitted to the responses of the complex-cell models to sine-grating stimuli (see S1 Appendix for details of the fitting operation). Prince et al. used both the phase and position of a Gabor function fitted to complex-cell responses[8], the sine-grating stimulus used in this analysis is cyclical so a sine wave alone is sufficient. The phase of the response function is based on a cosine so we added π to the phase of the sine wave function. The results are shown in Fig 8. For strongly binocularly selective complex-cell models (DDI > 0.6) we found only 0 and π phase responses corresponding to Tuned Excitatory and Tuned Inhibitory model respectively. 62% (±13.9% 95% Confidence Intervals) of complex-cell complements are Tuned Excitatory, 37% (±17.74% 95% Confidence Intervals) were Tuned Inhibitory. Other phases, corresponding to Near and Far cells were only found at low DDI i.e. in models that were not particularly tuned to detect disparity.

### Max pooling models

An alternative to the quadrature pair energy model is the max pooling model. This model is heavily used in computer vision [49–51]. The max pooling model has been suggested as a possible alternative to the energy model in understanding complex cell responses [52, 53]. In our implementation of the max pooling model, the sub-units are learned using ISA as before and the responses of the linear model are combined by taking the maximum absolute response of the two sub-units as the complex model response.

**Fig 9:**
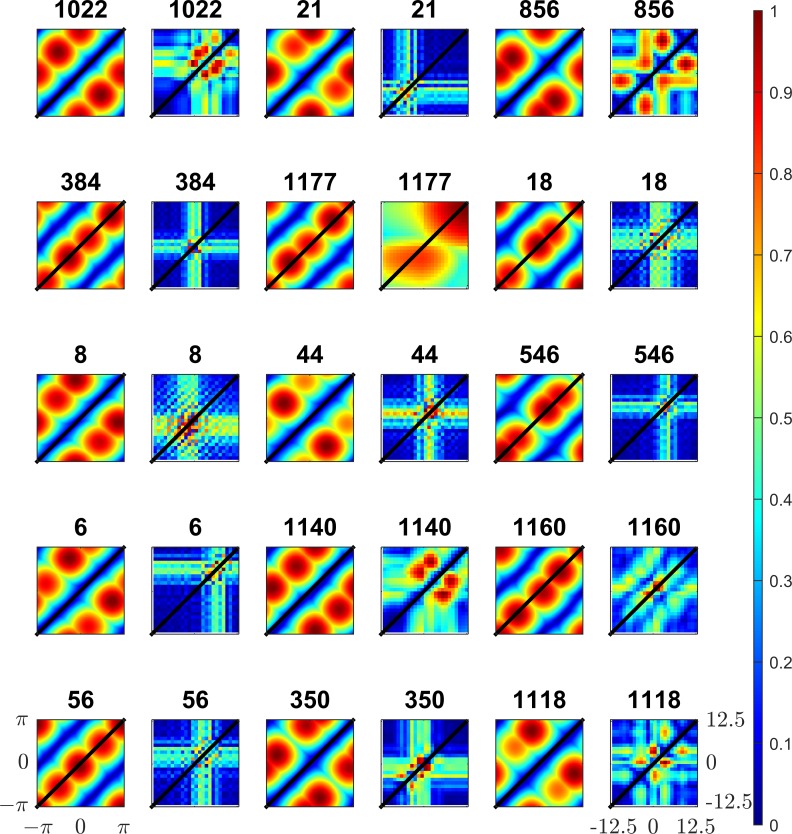
Responses of complex cell models using the max-pooling model to both sine-gratings and bar stimuli. Columns 1,3,5 show the responses of the models to sine-gratings stimuli, columns 2,4,6 show responses of the models in the odd columns to bar-stimuli. The models are chosen for their high DDI. As with the energy model (see Figs 4 and 6) the strongest responses lie on diagonals of equal disparity, however unlike the energy model the response function are less smooth, with greater range of responses exhibited on the diagonals of both the sine-grating responses and the bar stimuli responses.

**Fig 10:**
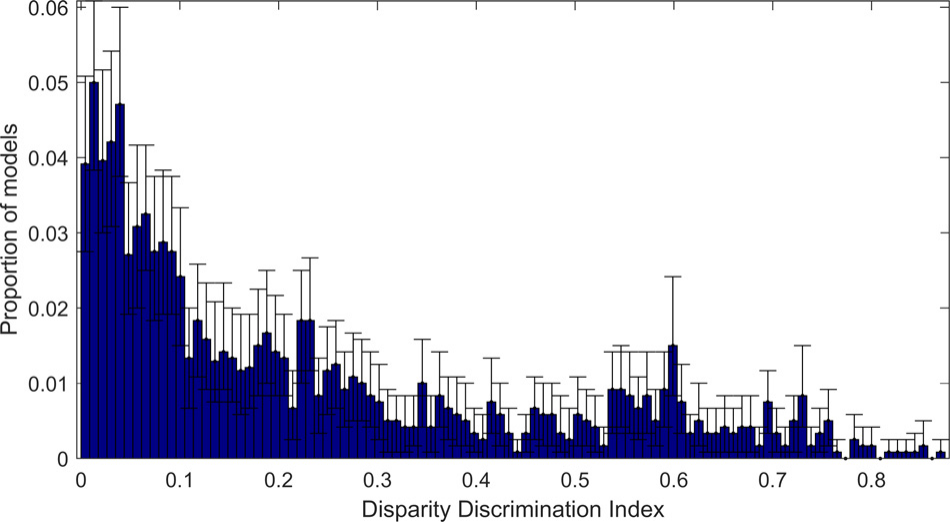
Distribution of DDI scores for max-pooled complex cell models. The distribution of DDI scores is bootstrapped 200 times to produce 95% Confidence Intervals (vertical black lines) and median scores (blue boxes) for each cell in a histogram. The max-pooling model exhibits a wide range of binocular disparities between 0 and 0.8743.

Fig 9 shows the responses of max pooling complex models to both sine-grating and bar stimuli. As the models are chosen for their high DDI most of the strong responses lie on points of equal disparity. As with the energy model max-pooled models can exhibit a high degree of binocular sensitivity. However, the response functions of the max-pooled model are less smooth compared to responses of the standard energy model (compare sine-grating responses of max-pooled models (Fig 9) with responses of the sum of squared responses model (Fig 6)). This indicates that the max pooling model is less phase invariant than the energy model. Fig 10 shows the distribution of DDI scores for the max-pooling model. The energy and max-pooling models exhibit similarly wide ranges of binocular disparity sensitivities. However the highest DDI score for the max pooling model was 0.87 compared to 0.95 (see Fig 7) for the energy model.

**Fig 11:**
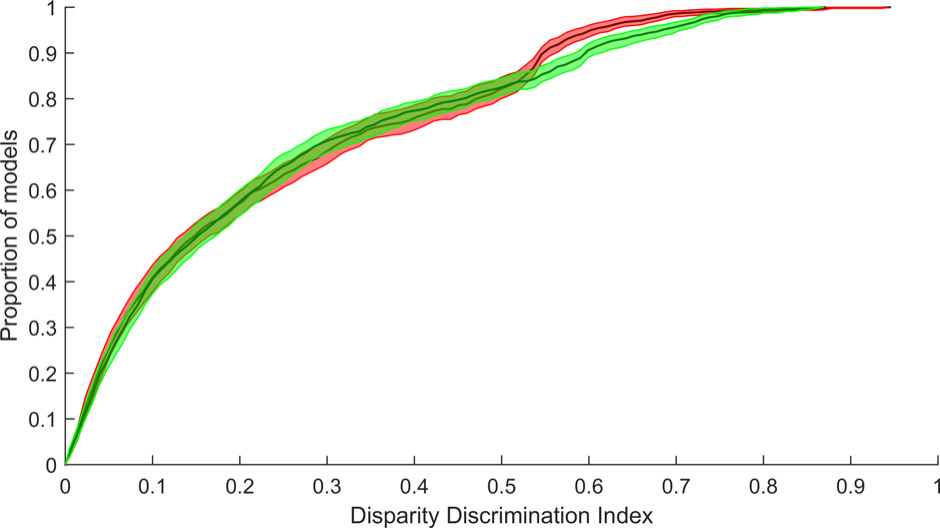
Comparison of cumulative distributions of DDI for both max-pooled and energy model complex cell models. The cumulative distribution for the max-pooled model is shown in green and the cumulative distribution of the energy model DDI is shown in red. Confidence intervals are generated using 200 bootstrapped distributions, the median of the distributions is shown as a black line in both models, the 95% CI shown as a green (max-pooled) or red (energy model) band. In the lower half for the distribution (DDI values below 0.5) no significant deviation is found between the two distributions. In the upper half a marked bias emerges, with the energy model significantly higher than the max-pooled model.

When the distributions of DDI for both max-pooled and energy model complex-cell components are compared (Fig 11), the distributions are found to be remarkably similar below a DDI of 0.5. Above a DDI of 0.5 a significant separation occurs due to the energy model’s bias to values around 0.55. In this area the energy model is under-performing compared to the max-pooling model, as the energy model is biased towards lower DDI compared to the max-pooling model.

## Discussion

Theoretical complex cell models such as the binocular energy model [3], and derivatives of this model, calculate disparity by combining the squared responses of simple cell models with Gabor-shaped receptive fields in quadrature phase. Quadrature pairs of Gabor functions are by definition orthogonal (decorrelated), so the orthogonality constraint applied by ISA to the sub-spaces makes sense in a binocular context. Assuming the signal (or part thereof) has the same frequency and orientation as the carrier wave of the Gabor function, the well-known Fourier shift theory holds that the result of convolving the signal with the Gabor function will be the cosine of the phase difference between the signal and Gabor function. As a consequence, two Gabor functions in quadrature phase are sufficient to calculate the local phase of a signal [10], and two pairs of Gabor functions in quadrature phase in each eye are sufficient to calculate the disparity between two signals of equal frequency and orientation in each eye [4]. This combination of components implies that the joint distribution of responses is polar and equal in all directions. Such a distribution can be considered to lie on a space described by an *L*_2_ norm.

Assumptions about the interaction of complex cell sub-units underpin current analyses of complex-cell physiology. Most studies have assumed a summative interaction, where the responses of linear-non-linear sub-units are added (excitatory interaction) or subtracted (inhibitory interaction) [45, 54]. A substantial amount of research into complex-cells in the visual cortex suggest that they can be modelled as being built from simple-cell-like sub-units[36, 43](or see [31, 32] for an overview). More recent research has been able to characterise these sub-units based on the assumption that the sub-units are orthogonal and follow a Gaussian distribution [27, 28, 55]. These sub-unit profiles resembled the well-known Gabor-like profiles of simple-cells. These are the same assumptions that underlie Independent Subspace Analysis, and component subspaces exhibiting phase invariant-like structures have been found in analyses of monocular images [15] using ISA.

ISA formulates the problem of unsupervised learning of complex-cell like models as a sub-space sparsity maximisation problem[16]. An alternative formulation often attributed to Klopf [56] (as reported by [57]) is a slowness or stability criterion, where the aim is to minimise variation over time of the complex-cells’ responses [57–59]. Clearly as the slowness criteria applies to the temporal domain it is not directly applicable to the analysis of static binocular images, however the criterion is of interest to research into complex-cells. The slowness criterion has been adapted for complex-cells by e.g. Kayser et al.[58] and Berkes and Wiskott[59] Although Wiskott’s slow feature analysis[60] has been shown to be an alternative form of second-order ICA[61] it differs substantially from higher-order independent components analysis that forms the basis for ISA[15, 62]. By emphasising sparseness ISA learns features with substantially smaller receptive fields than the slowness criteria[62].

Independent Subspace Analysis combines a Gaussian distributed *L*_2_ norm on responses with a subspace with a term that maximises super-Gaussianity of responses between subspaces. If the underlying structure of the observed data can be described using sparse subspaces, this model will produce sparse responses between subspaces. By training such a model on a set of binocular image patches, we can produce a set of components that decompose the image patches into a set of linear components. These components are grouped into subspaces.

Using the components within a subspace, we can build a complex-cell model. Each individual component is used to create a single linear-non-linear single-cell model, the responses of which are combined to create the complex-cell. Using binocular sine-gratings varying in phase across each left/right view we explored the range of responses of each complex-cell model to varying disparity across phases.

We found that the complex-cell models learned using ISA exhibited a wide range of binocular responses. The models produced were biased towards low values of DDI, with only a minority exhibiting strong binocular disparity discrimination ability. In many respects this is unsurprising; the ISA model is expected to approximately span all image features in the image patches, binocular disparity is only one aspect of the image patches that the model needs to cover. Other complex-cell models have been found that are specifically monocular in their responses or tuned to detect high specific phase combinations in each eye. However, when energy model components were examined, the majority of components were found to have low DDI. This may be due to the measure used to define binocular disparity discriminability being dependent on signal phase alone; this measure will fail to capture models where mixtures of one or more of phase, receptive field position, frequency or orientation are combined to produce binocular disparity tuned models. Neurons that combine phase and position disparity are prevalent in the visual cortices of cats and primates [8, 9, 63] and have been found to be prevalent in statistical analysis of binocular image pairs [21]. The proportion of models with high disparity discrimination tunings may therefore be under-estimated using this metric.

The model produced a sub-set of complex-cell models that were strongly tuned to binocular disparity and could therefore be utilised in the perception of stereoscopic depth. Complex-cells not tuned to detect binocular disparity may be specialised towards detection of other features in the binocular image set; e.g. purely monocular features, orientation or frequency invariance, or significant non-invariant features. The discovery that a sub-set of complex-cell models are highly tuned for disparity while the majority are not indicates a tendency towards specialisation in complex-cell models learned using the ISA model.

The symmetry of variations in responses of neurons to stimuli of varying disparity has been used by a number of authors to classify these cells in terms of their binocular responses[47]. Most authors have found a strong bias towards even symmetric disparity response function (TE/TI) in neurons compared to odd symmetric disparity responses (Near/Far)[8, 47, 48]. A representative result from Prince et al. found 38% TE, 16%TI, 21% Near and 25% Far[8]. In our analysis we found no evidence of Near or Far tuning in complex cell models with high DDI. Complex cell models tuned to detect disparity only exhibited 0 and π phase response functions corresponding to the Tuned Excitatory and Tuned Inhibitory labels. We also found no evidence for a bias towards either TE or TI type complex cell models. These models are tuned to detect disparity exclusively along the horoptor. This is consistent with earlier results of Hunter and Hibbard [21] who found that single cell binocular models learned using Independent Component Analysis[18] were highly tuned to detect disparities at integer and half-integer multiples of the wavelength of the simple-cell, that is Tuned Excitatory (integer multiples) and Tuned Inhibitory (half-integer multiples) cells. The finding that ISA learns disparity tuned complex cell models tuned exclusively to TE and TI types is inconsistent with physiology, where TE and TI types together accounts for between 50% and 60% of cells recorded[8, 47, 48] and a clear bias towards TE cells exists. Similarly the ISA results do not match previous statistical analysis of range data [64] and image analysis[37]. Although no Near or Far type neurons were found, the ratio of TE and TI cells fell within range of values predicted by both physiology[8, 47, 48], range data[64] and image statistics[37].

Why both ICA[21] and ISA fail to produce models with disparity distributions that match either physiology or known disparity distributions is an open question. It is too early to suggest that sparsity is the culprit as other sparse coding algorithms e.g. *L*_1_ [23] may better account for observed disparity distributions. ISA attempts to provide an efficient coding of the binocular image patches, the number of components that can be learned is limited and ISA prioritises components that maximally explain the data. Both ISA and ICA require that components are orthogonal. While this makes computation easier, it imposes an unrealistic restriction on the shape of the components. Simple zero-disparity components arranged in quadrature phase form an orthogonal basis but does not fully span the space of disparities, a non-orthogonal model may have the freedom to fully cover the range of possible disparities.

Although the energy model is capable of producing a more refined output than the max-pooling model we found that the max-pooling model was also capable of producing complex cells that closely matched the expectation of a neuron tuned to detect disparity in a phase invariance manner. The distribution of DDI was extremely similar for both models, with the energy model being slightly biased toward lower DDIs at the upper end of the scale. This is surprising as the standard energy model is capable of providing significantly higher DDI than max-pooling due to the smoothness of the response function across phase. In our results near perfect quadrature pairs, although rare, were found (see Fig 5) and the maximum DDI found for the energy model was significantly high than for max-pooling.

At low DDI, the complex cell models are not tuned for binocular disparity, and so the choice of a combination function may not be so important. At higher DDI, max-pooling actually outperforms the energy model; only at the very highest DDI values do the advantages of the energy model become apparent. Although the max-pooling model is not capable of the refined results of the energy model this may not be necessary for providing a reasonable generative approximation of the image patch set. Max-pooling may be an effective ‘poor-man’s’ alternative to the energy model in scenarios where the energy is costly to compute.

## Limitations of Our Analysis

The model of simple linear-non-linear models used here has a substantial limitation in that it does not allow for half-wave rectification. Half-wave rectification of sub-unit responses has been found to play a substantial role in binocular visual processing. For example, Read and Cumming [65] found evidence for both inhibitory and excitatory combinations of binocular stimuli post-filtering and rectification.

In the interests of presenting an easily understood model we have, in common with other authors, presented simple-cells versus complex cells as a simple dichotomy. It is well known in the complex cell literature that cells in V1 in fact exhibit a wide range of ‘complexity’. Movshon et al. found that the elongation of receptive fields lay on a continuum[45]. Using spike triggered covariance, numerous authors have shown that even so called simple-cells have non-linear receptive fields [33, 55]. Again using STC authors have found that the number of relative weights of both excitatory and inhibitory sub-units varies considerably in any population of neurons[33, 55, 66].

Our model fixed the number of sub-units to two. This is a somewhat arbitrary number chosen mainly due to the similarity with the standard energy model. The number of sub-units of complex cells has been found to be highly variable in both physiological recordings [33, 55] and in statistical models of natural images [67].

## Relationship to Other Models of Physiology

Previous work has applied ICA to natural binocular images [20, 21]. This has resulted in components bearing a number of similarities to binocular simple cells in the visual cortex. Binocular independent components tend to be Gabor-like, with very similar orientation and frequency tuning for the two eyes, and a range of position and phase disparity tunings. However, there were also a number of differences. In particular, the distribution of phase-disparity tunings did not match that found in the visual cortex. While phase disparities are strongly peaked around zero in cortical cells, independent components also showed a marked peak for anti-phase components [21]. This distribution was also found with ISA.

ICA is limited to modelling the first-stage of binocular encoding. A consequence of this is that the responses of the resulting components are dependent on the monocular phases of the input stimulus. This limitation, as a model of an ideal disparity detector, is shared by other approaches to understanding binocular encoding, such as Li and Atick’s model of binocular summation and differencing channels [68]. These models focus on the efficient encoding of binocular images, rather than on the estimation of disparity.

This limitation is overcome in the binocular energy model by summing the squared responses of filters with quadrature phase tuning. This specific phase relationship is not necessary to achieve phase invariance, as the summation of responses across a population of filters with differing phase tuning can be used to produce a phase-invariant response [3]. There is thus a direct link between the phase-invariance achieved by ISA, and that required for an ideal disparity detector. Burge et al showed that a model approximating optimal disparity estimation can be created by summing the responses of linear filters, following static thresholding and squaring non-linearities[30].

## Concluding Remarks

Our work has shown that an ISA model applied to binocular natural images produces complex cell models, a subset of which fit the criteria of an ideal disparity detector [4]. In particular, a subset of the models generated by ISA displayed high levels of local position and phase invariance, resulting in a response that was constant for all stimulus positions within the receptive field. Responses of the model to anti-correlated stimuli are also zero or near to zero in complex cell models with high DDI, as required by an ideal disparity detector.

## Supporting Information

S1 Appendix Binocular disparity discrimination index. Description of the algorithm to calculate the binocular disparity discrimination index from model responses.
(DOCX)

S1 Fig Calculation of Disparity Discrimination Index on hypothetical data. The DDI measures the range of a sine-grating function fitted to the responses and compares it to the maximum of sine-grating.
(TIF)

## References

[1] Tyler CW, Kontsevich LL. Mechanisms of stereoscopic processing: stereoattention and surface perception in depth reconstruction. Perception-London. 1995;24(1):127–54. 7617422 10.1068/p240127

[2] Banks MS, Gepshtein S, Landy MS. Why is spatial stereoresolution so low? The Journal of Neuroscience. 2004;24(9):2077–89. 14999059 10.1523/JNEUROSCI.3852-02.2004 PMC6730432

[3] Fleet DJ, Wagner H, Heeger DJ. Neural encoding of binocular disparity: energy models, position shifts and phase shifts. Vision Research. 1996;36(12):1839–57. 8759452 10.1016/0042-6989(95)00313-4

[4] Ohzawa I, DeAngelis GC, Freeman RD. Stereoscopic depth discrimination in the visual cortex: neurons ideally suited as disparity detectors. Science. 1990;249(4972):1037–41. 2396096 10.1126/science.2396096

[5] Marr D, Poggio T. A computational theory of human stereo vision. Proc R Soc Lond B. 1979;204:301–28. 37518 10.1098/rspb.1979.0029

[6] Watanabe O. Stereo transparency in ambiguous stereograms generated by overlapping two identical dot patterns. Journal of Vision. 2009;9(12):24 10.1167/9.12.24 20053115

[7] Ohzawa I, Freeman RD. The binocular organization of complex cells in the cat's visual cortex. Journal of Neurophysiology. 1986;56(1):243–59. 3746399 10.1152/jn.1986.56.1.243

[8] Prince SJP, Cumming BG, Parker AJ. Range and mechanism of encoding of horizontal disparity in macaque V1. J Neurophysiol. 2002;87:209–21. 11784743 10.1152/jn.00466.2000

[9] Prince SJP, Pointon A, Cumming BG, Parker A. Quantitative analysis of the responses of V1 neurons to horizontal disparity in dynamic random-dot stereograms. Journal of Neurophysiology. 2002;87(1):191–208. 11784742 10.1152/jn.00465.2000

[10] Adelson EH, Bergen JR. Spatiotemporal energy models for the perception of motion. J Opt Soc Am A. 1985;2(2):284–99. 3973762 10.1364/josaa.2.000284

[11] Qian N, Zhu Y. Physiological computation of binocular disparity. Vision Research. 1997;37(13):1811–27. 9274767 10.1016/s0042-6989(96)00331-8

[12] Barlow HB. Possible principles underlying the transformation of sensory messages. Sensory communication. 1961:217–34.

[13] Olshausen BA, Field DJ. Natural image statistics and efficient coding*. Network: computation in neural systems. 1996;7(2):333–9. 10.1088/0954-898X/7/2/014 16754394

[14] Hyvärinen A, Hurri J, Hoyer PO. Natural Image Statistics: A probabilistic approach to early computational vision: Springer-Verlag New York Inc; 2009.

[15] Hyvärinen A, Hoyer P. Emergence of phase-and shift-invariant features by decomposition of natural images into independent feature subspaces. Neural Computation. 2000;12(7):1705–20. 10935923 10.1162/089976600300015312

[16] Okajima K. Binocular disparity encoding cells generated through an infomax based learning algorithm. Neural Netw. 2004;17(7):953–62. 10.1016/j.neunet.2004.02.004 15312838

[17] Bell AJ, Sejnowski TJ. An information-maximization approach to blind separation and blind deconvolution. Neural Computation. 1995;7(6):1129–59. 7584893 10.1162/neco.1995.7.6.1129

[18] Hyvarinen A. Fast and robust fixed-point algorithms for independent component analysis. Neural Networks, IEEE Transactions on. 1999;10(3):626–34. 10.1109/72.761722 18252563

[19] Ringach DL. Spatial structure and symmetry of simple-cell receptive fields in macaque primary visual cortex. Journal of neurophysiology. 2002;88(1):455–63. 12091567 10.1152/jn.2002.88.1.455

[20] Hoyer PO, Hyvärinen A. Independent component analysis applied to feature extraction from colour and stereo images. Network: computation in neural systems. 2000;11(3):191–210. 11014668

[21] Hunter DW, Hibbard PB. Distribution of independent components of binocular natural images. Journal of Vision. 2015;15(13):6–. 10.1167/15.13.6 26381837

[22] Anzai A, Ohzawa I, Freeman RD. Neural mechanisms underlying binocular fusion and stereopsis: position vs. phase. Proc Natl Acad Sci USA. 1997;94:5438–43. 9144256 10.1073/pnas.94.10.5438 PMC24697

[23] Olshausen BA, Field DJ. Sparse coding with an overcomplete basis set: A strategy employed by V1? Vision Research. 1997;37(23):3311–25. 9425546 10.1016/s0042-6989(97)00169-7

[24] Olshausen BA, Field DJ. How close are we to understanding V1? Neural Computation. 2005;17(8):1665–99. 15969914 10.1162/0899766054026639

[25] Olshausen BA, Cadieu CF, Warland DK, editors. Learning real and complex overcomplete representations from the statistics of natural images. SPIE Optical Engineering+ Applications; 2009: International Society for Optics and Photonics.

[26] Hyvärinen A, Hoyer P. Emergence of topography and complex cell properties from natural images using extensions of ICA. Advances in Neural Information Processing Systems. 2000;12:827–33.

[27] Pillow JW, Simoncelli EP. Dimensionality reduction in neural models: an information-theoretic generalization of spike-triggered average and covariance analysis. Journal of Vision. 2006;6(4):9. 10.1167/6.4.9 16889478

[28] Schwartz O, Pillow JW, Rust NC, Simoncelli EP. Spike-triggered neural characterization. Journal of Vision. 2006;6(4):13. 10.1167/6.4.13 16889482

[29] Sasaki KS, Tabuchi Y, Ohzawa I. Complex cells in the cat striate cortex have multiple disparity detectors in the three-dimensional binocular receptive fields. The Journal of Neuroscience. 2010;30(41):13826–37. 10.1523/JNEUROSCI.1135-10.2010 20943923 PMC6633723

[30] Burge J, Geisler WS. Optimal disparity estimation in natural stereo images. Journal of vision. 2014;14(2):1 10.1167/14.2.1 24492596 PMC3912897

[31] Anzai A, Ohzawa I, Freeman RD. Neural mechanisms for processing binocular information II. Complex cells. Journal of Neurophysiology. 1999;82(2):909–24. 10444686 10.1152/jn.1999.82.2.909

[32] Carandini M. Do we know what the early visual system does? J Neurosci. 2005;25:10577–97. 16291931 10.1523/JNEUROSCI.3726-05.2005 PMC6725861

[33] Chen X, Han F, Poo M-m, Dan Y. Excitatory and suppressive receptive field subunits in awake monkey primary visual cortex (V1). Proceedings of the National Academy of Sciences. 2007;104(48):19120–5. 10.1073/pnas.0706938104 PMC2141918 18006658

[34] Haefner RM, Cumming BG. Adaptation to natural binocular disparities in primate V1 explained by a generalized energy model. Neuron. 2008;57(1):147–58. 10.1016/j.neuron.2007.10.042 18184571 PMC2344156

[35] Ohzawa I, Deangelis GC, Freeman RD. Encoding of binocular disparity by complex cells in the cat's visual cortex. Journal of Neurophysiology. 1997;77(6):2879–909. 9212245 10.1152/jn.1997.77.6.2879

[36] Szulborski RG, Palmer LA. The two-dimensional spatial structure of nonlinear subunits in the receptive fields of complex cells. Vision Research. 1990;30(2):249–54. 2309459 10.1016/0042-6989(90)90040-r

[37] Hibbard PB. Binocular energy responses to natural images. Vision Research. 2008;48(12):1427–39. 10.1016/j.visres.2008.03.013 18456305

[38] Bouguet J-Y. Camera calibration toolbox for matlab. 2004.

[39] Hong G, Luo MR, Rhodes PA. A study of digital camera colorimetric characterisation based on polynomial modelling. 2001.

[40] Srinivasan M, Laughlin S, Dubs A. Predictive coding: a fresh view of inhibition in the retina. Proceedings of the Royal Society of London Series B Biological Sciences. 1982;216(1205):427–59. 10.1098/rspb.1982.0085 6129637

[41] Atick JJ, Redlich AN. What does the retina know about natural scenes? Neural Computation. 1992;4(2):196–210.

[42] Laughlin S. A simple coding procedure enhances a neuron's information capacity. Z Naturforsch C. 1981;36:910–2. 7303823

[43] Heggelund P. Receptive field organization of simple cells in cat striate cortex. Experimental Brain Research. 1981;42(1):89–98. 7215513 10.1007/BF00235733

[44] Hubel DH, Wiesel TN. Receptive fields, binocular interaction and functional architecture in the cat's visual cortex. The Journal of physiology. 1962;160(1):106–54. 14449617 10.1113/jphysiol.1962.sp006837 PMC1359523

[45] Movshon J, Thompson I, Tolhurst D. Receptive field organization of complex cells in the cat's striate cortex. The Journal of physiology. 1978;283(1):79–99. 722592 10.1113/jphysiol.1978.sp012489 PMC1282766

[46] Movshon J, Thompson I, Tolhurst D. Spatial summation in the receptive fields of simple cells in the cat's striate cortex. The Journal of physiology. 1978;283(1):53–77. 722589 10.1113/jphysiol.1978.sp012488 PMC1282765

[47] Poggio GF, Gonzalez F, Krause F. Stereoscopic mechanisms in monkey visual cortex: binocular correlation and disparity selectivity. The Journal of Neuroscience. 1988;8(12):4531–50. 3199191 10.1523/JNEUROSCI.08-12-04531.1988 PMC6569547

[48] Durand J-B, Celebrini S, Trotter Y. Neural bases of stereopsis across visual field of the alert macaque monkey. Cerebral Cortex. 2007;17(6):1260–73. 16908495 10.1093/cercor/bhl050

[49] Jarrett K, Kavukcuoglu K, Ranzato MA, LeCun Y. What is the best multi-stage architecture for object recognition? Proc IEEE Int Conf Comput Vis. 2009.

[50] LeCun Y, Bengio Y, Hinton G. Deep learning. Nature. 2015;521(7553):436–44. 10.1038/nature14539 26017442

[51] Weng J, Ahuja N, Huang TS, editors. Cresceptron: a self-organizing neural network which grows adaptively. Neural Networks, 1992 IJCNN, International Joint Conference on; 1992: IEEE.

[52] Riesenhuber M, Poggio T. Hierarchical models of object recognition in cortex. Nature Neurosci. 1999;2:1019–25. 10526343 10.1038/14819

[53] Hansard M, Horaud R. A differential model of the complex cell. Neural Computation. 2011;23(9):2324–57. 10.1162/NECO_a_00163 21671791

[54] Pettigrew J, Nikara T, Bishop P. Binocular interaction on single units in cat striate cortex: simultaneous stimulation by single moving slit with receptive fields in correspondence. Experimental Brain Research. 1968;6(4):391–410. 5721767 10.1007/BF00233186

[55] Rust NC, Schwartz O, Movshon JA, Simoncelli EP. Spatiotemporal elements of macaque v1 receptive fields. Neuron. 2005;46(6):945–56. 15953422 10.1016/j.neuron.2005.05.021

[56] Klopf AH. The hedonistic neuron: a theory of memory, learning, and intelligence: Toxicology-Sci; 1982.

[57] Földiák P. Learning invariance from transformation sequences. Neural Computation. 1991;3(2):194–200. 10.1162/neco.1991.3.2.194 31167302

[58] Kayser C, Einhäuser W, Dümmer O, König P, Körding K. Extracting slow subspaces from natural videos leads to complex cells Artificial Neural Networks—ICANN 2001: Springer; 2001 p. 1075–80.

[59] Berkes P, Wiskott L. Slow feature analysis yields a rich repertoire of complex cell properties. Journal of Vision. 2005;5(6):9. 10.1167/5.6.9 16097870

[60] Wiskott L, Sejnowski TJ. Slow feature analysis: Unsupervised learning of invariances. Neural Computation. 2002;14(4):715–70. 11936959 10.1162/089976602317318938

[61] Blaschke T, Berkes P, Wiskott L. What is the relation between slow feature analysis and independent component analysis? Neural Computation. 2006;18(10):2495–508. 16907634 10.1162/neco.2006.18.10.2495

[62] Lies J-P, Häfner RM, Bethge M, Lucke J. Slowness and sparseness have diverging effects on complex cell learning. PLoS Computational Biology. 2014;10(3):e1003468 10.1371/journal.pcbi.1003468 24603197 PMC3945087

[63] Read JCA, Cumming BG. Sensors for impossible stimuli may solve the stereo correspondence problem. Nature neuroscience. 2007;10(10):1322–8. 17828262 10.1038/nn1951 PMC2075086

[64] Liu Y, Bovik AC, Cormack LK. Disparity statistics in natural scenes. Journal of Vision. 2008;8(11):19 10.1167/8.11.19 18831613

[65] Read JCA, Cumming BG. Ocular dominance predicts neither strength nor class of disparity selectivity with random-dot stimuli in primate V1. J Neurophysiol. 2004;91:1271–81. 14523074 10.1152/jn.00588.2003 PMC1410815

[66] Fournier J, Monier C, Levy M, Marre O, Sári K, Kisvárday ZF, et al Hidden Complexity of Synaptic Receptive Fields in Cat V1. The Journal of Neuroscience. 2014;34(16):5515–28. 10.1523/JNEUROSCI.0474-13.2014 24741042 PMC6608221

[67] Gutmann MU, Hyvärinen A. A three-layer model of natural image statistics. Journal of Physiology-Paris. 2013;107(5):369–98. 10.1016/j.jphysparis.2013.01.001 23369823

[68] Li Z, Atick JJ. Efficient stereo coding in the multiscale representation*. Network: computation in neural systems. 1994;5(2):157–74.

