# Supplementary material for: Ideal Binocular Disparity Detectors Learned Using Independent Subspace Analysis on Binocular Natural Image Pairs
Source: PLoS One. 2016 Mar 16;11(3):e0150117. doi: 10.1371/journal.pone.0150117 (PMC4794214; doi:10.1371/journal.pone.0150117)
Supplement: S1 Appendix — Description of the algorithm to calculate the binocular disparity discrimination index from model responses. (DOCX) [file pone.0150117.s001.docx]

# S1 Appendix. Binocular disparity discrimination index

The binocular disparity discrimination index (DDI) introduced by Prince et al. (18) is a measure of the change in response due to a change in disparity. Cells that are strongly selective to disparity will exhibit a large change in response when disparity is varied, while disparity invariant cells will exhibit a narrow range of responses across disparity.

The disparity discrimination index compares an idealised disparity function, composed of a sine-wave function to the observed data. The idealised disparity function is first fitted to the observed data, the index is then the ratio between the modulation of the idealized disparity function across varying disparity and the modulation of the idealized disparity function plus the fitting error. This is visualised in Fig 12

The DDI was calculated using the responses of models to sine-grating stimuli. In order to reduce the influence of outliers on the extrema a smoothing function was fitted to the data and the range of responses taken from the range of fitted functions; this is diagrammed in S1 Fig. A sine-wave function was fitted to the responses to a modelled complex cell at each disparity.

|  | $r_{i}=\alpha\sin\left( \beta d_{i}+\gamma\right)+\delta+\epsilon$ | (9) |
| --- | --- | --- |

Where $d_{i}$ is the disparity at each location $i$ in the response map and $r_{i}$ the response at that location and $\epsilon\sim N(0,\sigma)$ is the residuals. By rearranging equation 9 the residuals become $\epsilon=r-\alpha\sin\left( \beta d+\gamma\right)-\delta$ An approximation of the function over all responses $\boldsymbol{r=}\left\{ r_{1},r_{2},\ldots,r_{n} \right\}$ and associated disparities $\boldsymbol{d}=\left\{ d_{1},d_{2},\ldots,d_{n} \right\}$is found using standard error minimisation techniques.

|  | $\min_{\alpha,\beta,\gamma,\delta} \left( \boldsymbol{r}-\alpha\sin\left( \beta\boldsymbol{d}+\gamma\right)-\delta\right)^{2}$ | (10) |
| --- | --- | --- |

We minimised equation 10 for $\alpha,\beta,\gamma,\delta$ using Matlab’s curve fitting toolbox (68). We now have an equation for describing the estimated disparity response curve to a particular disparity:

|  | $f\left( d_{i} \vert\alpha,\beta,\gamma,\delta\right)=\alpha\sin\left( \beta d_{i}+\gamma\right)+\delta$ | (11) |
| --- | --- | --- |

The DDI for each complex model was calculated as:

|  | $ddi= \frac{\mathrm{range}(f\left( \boldsymbol{d} \vert\alpha,\beta,\gamma,\delta\right))}{\mathrm{range}(f\left( \boldsymbol{d} \vert\alpha,\beta,\gamma,\delta\right)+2\epsilon)}$ | (12) |
| --- | --- | --- |

Where $\mathrm{range}\left( f\left( \boldsymbol{d} | \alpha,\beta,\gamma,\delta\right) \right)$ is the range of values of the sine function over all generated disparities $\boldsymbol{d}$. Thus the DDI is the range of responses modulated by the extent of noise in the smoothing function. The DDI ranges from 0 to 1. A DDI of 1 indicates that a sine-wave function fully explains the data and the model is fully disparity selective independent of phase. A zero DDI indicates that the model is fully explained by noise and is not selective to disparity.
