# Supplementary figures and images for: Ideal Binocular Disparity Detectors Learned Using Independent Subspace Analysis on Binocular Natural Image Pairs
Source: PLoS One. 2016 Mar 16;11(3):e0150117. doi: 10.1371/journal.pone.0150117 (PMC4794214; doi:10.1371/journal.pone.0150117)

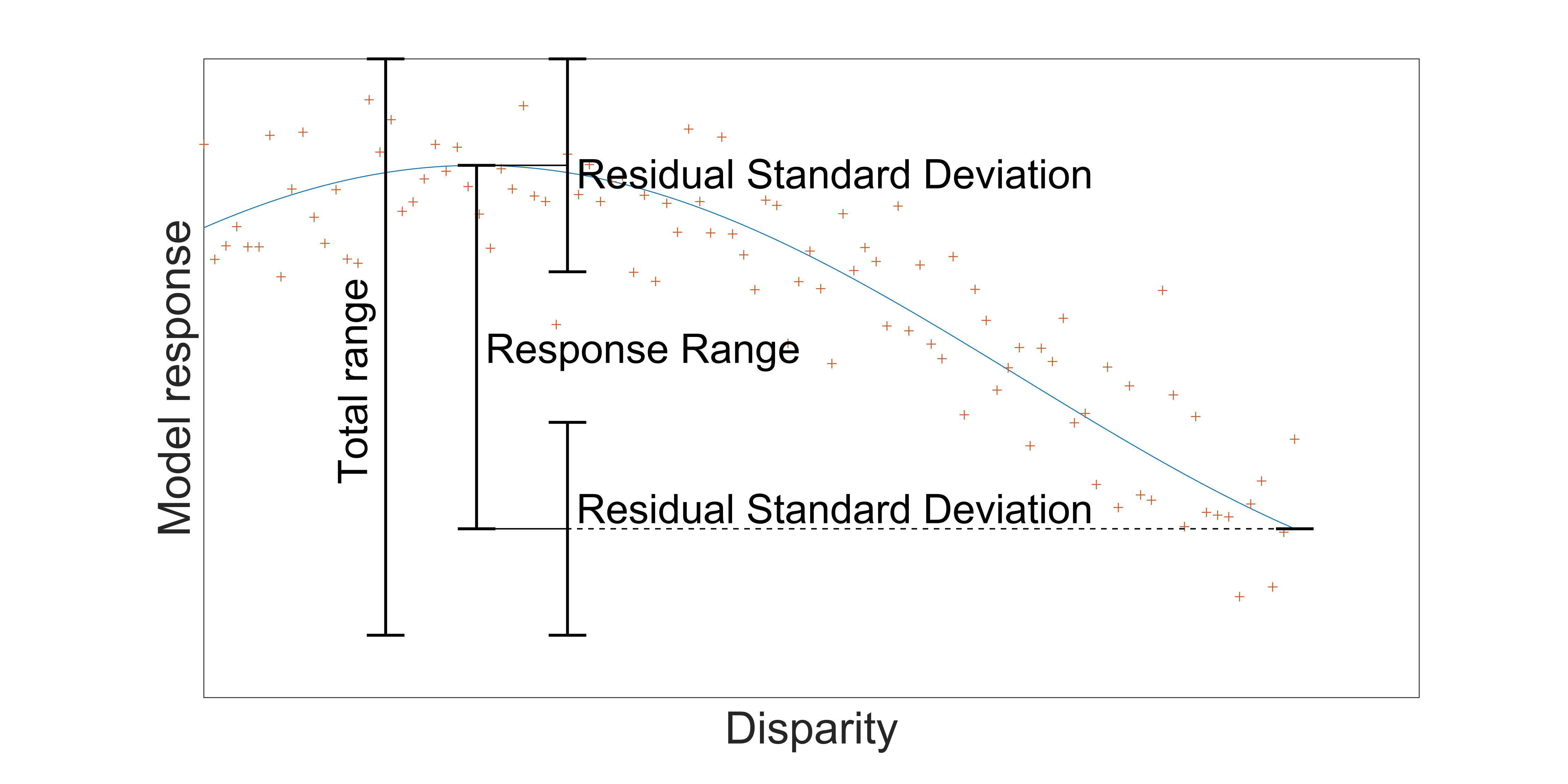

Supplement: S1 Fig — The DDI measures the range of a sine-grating function fitted to the responses and compares it to the maximum of sine-grating. (TIF) [file pone.0150117.s002.tif]
